# Moderate plasma dilution using artificial plasma expanders shifts the haemostatic balance to hypercoagulation

**DOI:** 10.1038/s41598-017-00927-w

**Published:** 2017-04-12

**Authors:** Elena I. Sinauridze, Alexander S. Gorbatenko, Elena A. Seregina, Elena N. Lipets, Fazoil I. Ataullakhanov

**Affiliations:** 1 National Scientific and Practical Centre of Pediatric Hematology, Oncology and Immunology named after Dmitry Rogachev, Russian Ministry of Health, Samory Mashela str. 1, GSP-7, Moscow, 117198 Russia; 2 Center for Theoretical Problems of Physicochemical Pharmacology, Russian Academy of Sciences, Kosygina str. 4, Moscow, 119334 Russia; 3 National Research Center for Hematology, Ministry of Health, Novyi Zykovskii pr. 4, Moscow, 125167 Russia; 4 Department of Physics, Moscow State University, Leninskie Gory 1, building 2, Moscow, 119991 Russia

## Abstract

Artificial plasma expanders (PEs) are widely used in modern transfusion medicine. PEs do not contain components of the coagulation system, so their infusion in large volumes causes haemodilution and affects haemostasis. However, the existing information on this effect is contradictory. We studied the effect of the very process of plasma dilution on coagulation and tested the hypothesis that moderate dilution with a PE should accelerate clotting owing to a decrease in concentration of coagulation inhibitors. The standard clotting times, a thrombin generation test, and the spatial rate of clot growth (test of thrombodynamics) were used to assess donor plasma diluted *in vitro* with various PEs. The pH value and Ca^+2^ concentration were maintained strictly constant in all samples. The effect of thrombin inhibitors on dilution-induced hypercoagulation was also examined. It was shown that coagulation was enhanced in plasma diluted up to 2.0–2.5-fold with any PE. This enhancement was due to the dilution of coagulation inhibitors in plasma. Their addition to plasma or PE could partially prevent the hypercoagulation shift.

## Introduction

Artificial plasma expanders (PEs) are the first-line choice among infusion solutions^1^ for restoration of the circulating fluid volume^2^. However, many contradictions exist regarding their impact on haemostasis; in particular, it remains unclear whether PE administration causes hyper- or hypo-changes of coagulation. None of the artificial PEs contain the components of the clotting system; therefore, large volumes of PE infusion inevitably lead to plasma dilution and to changes in the concentrations of coagulation components (haemodilution), thereby affecting the state of the coagulation system. Haemostatic disorder in trauma patients having received large-volume infusions of various blood products and/or PEs is a complex multifactorial process^2–4^. The result depends primarily on the type and volume of the transfused product as well as on some other factors related to trauma and haemodilution, such as the degree of reduction in the concentrations of the procoagulant factors^4^, counts of platelets^5, 6^ and red blood cells^7–10^, possible hypothermia^11–13^, and significant activation of coagulation in trauma patients due to the appearance of the extensive wound surface^14, 15^, etc. More detailed information can be found in reviews^2–4, 16^.

At the moment, there is no agreement among opinions about the magnitude of the effects that PE infusions exert on the plasma coagulation system. Even the sign of the effect is still in question. Microvascular and other bleeding events frequently observed after massive infusions make many clinicians intuitively believe that haemodilution should slow down coagulation because of the decreased concentrations of procoagulant factors and platelets^17–20^.

However, haemostasis is a finely balanced system with dozens of participants, not only procoagulants (prothrombin, fibrinogen, platelets, and others) but also anticoagulants (antithrombin III (AT), protein C, tissue factor pathway inhibitor (TFPI), etc.). It is impossible to predict the result of their simultaneous dilution from general considerations. Dilution can shift the balance in such a system to any side. This view has been supported by some clinical studies where the presence of a hypercoagulation state was shown after infusion of PEs^21–24^.

In this study, we focused on one fundamental issue: how the process of plasma dilution by itself (using any type of PE) affects the coagulation state. Given the data in the literature concerning the concentrations and mechanisms of the reactions for all the components of haemostasis^25–29^, we assumed that moderate plasma dilution would result in hypercoagulation primarily because of the decrease in the concentration of coagulation inhibitors. The aim of our study was to assess coagulation in plasma diluted with different PEs to test this hypothesis and to examine whether the addition of thrombin inhibitors to the system could prevent a dilution-induced shift to hypercoagulation.

## Results

### Standard clotting times in diluted plasma

**Figure 1:**
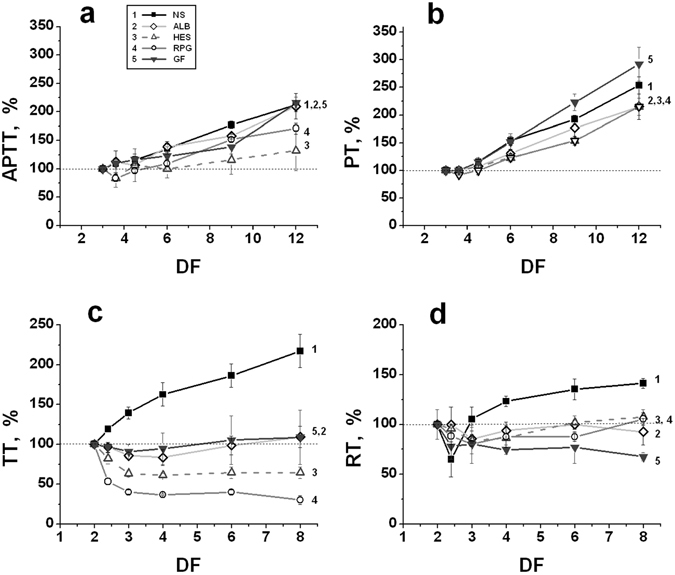
Standard clotting times of plasma diluted with various PEs *in vitro*. (**a**) – Activated partial thromboplastin time (APTT); (**b**) – prothrombin time (PT); (**c**) – thrombin time (TT); and (**d**) – recalcification time (RT). The following PEs were used for dilution: (1) – normal saline (NS); (2) – 10% solution of human albumin (ALB); (3) – 6% solution of hydroxyethyl starch 130/0.4 (Voluven, HES); (4) – 10% solution of dextran 40 (Reopolyglukin, RPG); (5) – 4% solution of succinylated gelatin (Gelofusine, GF). The value of each parameter in plasma without dilution was taken as 100%. The mean values ± standard deviation (SD) are presented (n = 6). DF (a dilution factor) characterizes the value of dilution and is equal to the ratio of the final volume of diluted plasma to its initial volume. DF shows the final dilution, taking into account the additional dilution of plasma during performance of the tests.

The averaged results of measurements for activated partial thromboplastin time (APTT), prothrombin time (PT), recalcification time (RT), and thrombin time (TT) in plasma diluted *in vitro* using different PEs are shown in Fig. 1.

The dilution degree in all the experiments was determined as a dilution factor (DF) that was equal to the ratio of the final volume of the diluted plasma to its initial volume. DF represents the total final dilution, taking into account the additional dilution of plasma during performance of the tests.

For all solutions, an increase in DF led to prolongation of PT and APTT (Fig. 1a,b), in good agreement with earlier published results^4, 13, 20, 25, 26, 30–34^. The behaviour of TT and RT was more complex. TT decreased in plasma diluted using any colloidal PE, but it increased after dilution with an isotonic solution of sodium chloride (normal saline, NS) (Fig. 1c). The reason for such behaviour cannot yet be explained. RT decreased in plasma that was diluted using any PE approximately 2.5-3 times. With a further increase in the dilution degree, RT began to increase (excepting Gelofusin), and TT ceased decreasing and remained at a constant level or very slowly increased (Fig. 1c,d).

### Thrombin generation in diluted plasma

**Figure 2:**
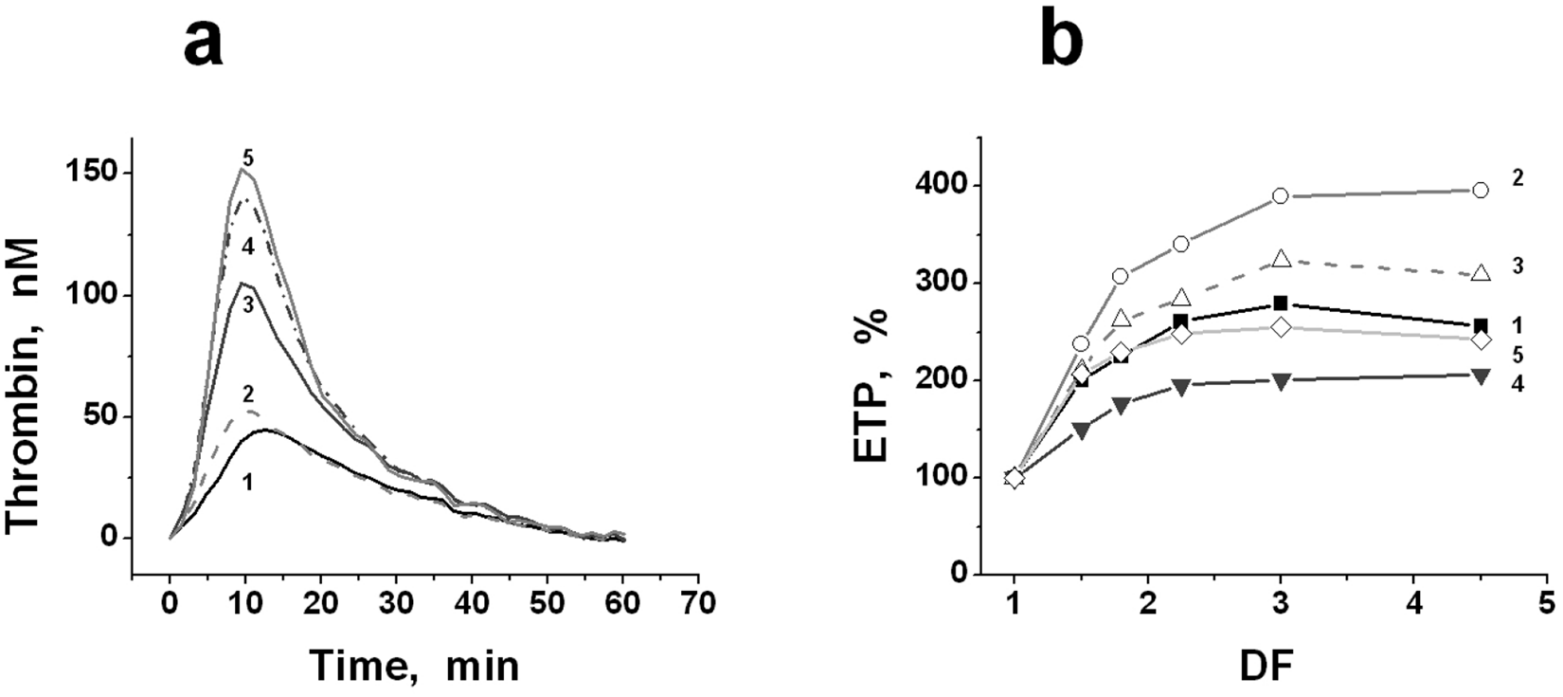
Thrombin generation in plasma diluted with PEs. (**a**) – The curves of thrombin generation in plasma diluted with NS (one of the typical experiments). The values of DF were equal to: (1) – 1.025 (plasma, practically undiluted); (2) – 1.5; (3) – 2; (4) – 3; (5) – 4. (**b**) – The typical dependencies of ETP (in %) versus DF obtained in one of the experiments for plasma diluted with different PEs (see legend to Fig. 1): (1) – NS; (2) – RPG; (3) –HES; (4) – GF; (5) – ALB. DF (a dilution factor) is equal to the ratio of the final volume of diluted plasma to its initial volume.

**Figure 3:**
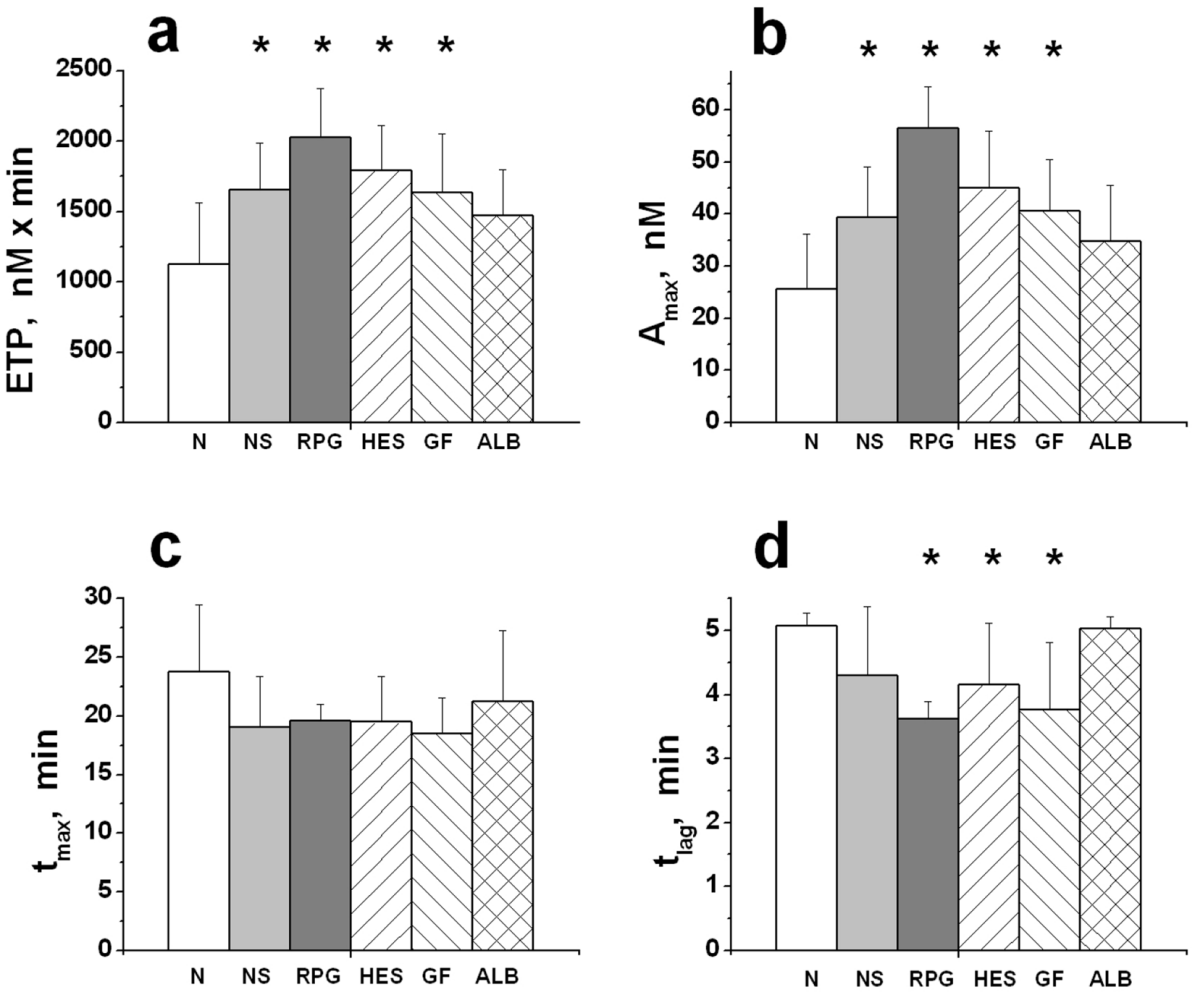
The parameters of TGT in undiluted plasma (N) and in plasma diluted with various PEs two-fold. (**a**) – The endogenous thrombin potential (ETP); (**b**) – the maximal concentration of thrombin in the sample (A_max_); (**c**) – time to maximal thrombin concentration (t_max_), and (**d**) – lag-time of thrombin generation (t_lag_). The following PEs were used for dilution of plasma (see legend to Fig. 1): NS, RPG, HES, GF, and ALB. The mean values ± standard deviation are shown (n = 8). *The values are significantly different from the corresponding values in undiluted plasma (ANOVA and paired t-test of Student, *P* < *0.05*).

The measurement of thrombin generation in plasma diluted with PEs showed that, in conditions of constant pH and a concentration of Ca^+2^-ions in all the samples, this generation increased with increasing DF (up to 2–2.5 times) for any PE. At higher DF, the thrombin generation began gradually to decrease, which was connected apparently to the strong decrease in concentration of the procoagulant factors. As an example, in Fig. 2, the curves of thrombin generation at different DFs are presented for plasma diluted with NS (Fig. 2a), and the values of endogenous thrombin potential (ETP) are shown as obtained in one of the typical experiments for plasma diluted using various PEs (Fig. 2b). Figure 3 shows the averaged results obtained in undiluted plasma and in plasma diluted two-fold with various PEs for all the parameters of the thrombin generation test (TGT): endogenous thrombin potential (area under the thrombin generation curve, ETP), maximal thrombin concentration in a sample (A_max_), time to this maximal concentration (t_max_), and lag-time to the beginning of accelerated thrombin formation (t_lag_), i.e., conditionally, the time when the thrombin concentration achieved 5 nM. The ETP values were increased significantly under plasma dilution with any PE excepting albumin (ALB), for which this increase was also observed but was not significant (Fig. 3a). The values of A_max_ also increased in a similar manner (Fig. 3b). The values of t_lag_ after the dilution of plasma with hydroxyethyl starch 130/0.4 (HES), reopolyglukin (RPG), and gelofusine (GF) were significantly shortened. For NS and ALB, the trend of the changes was the same; however, the differences were not significant (Fig. 3d). For all the investigated PEs, the values of t_max_ in diluted plasma had a trend towards shortening, which, however, was not significant (Fig. 3c). Thus, all the presented data indicate an increase of thrombin generation during moderate plasma dilution by various PEs in comparison to undiluted plasma.

### Changes in the concentration of coagulation factors and inhibitors with plasma dilution *in vitro*

**Figure 4:**
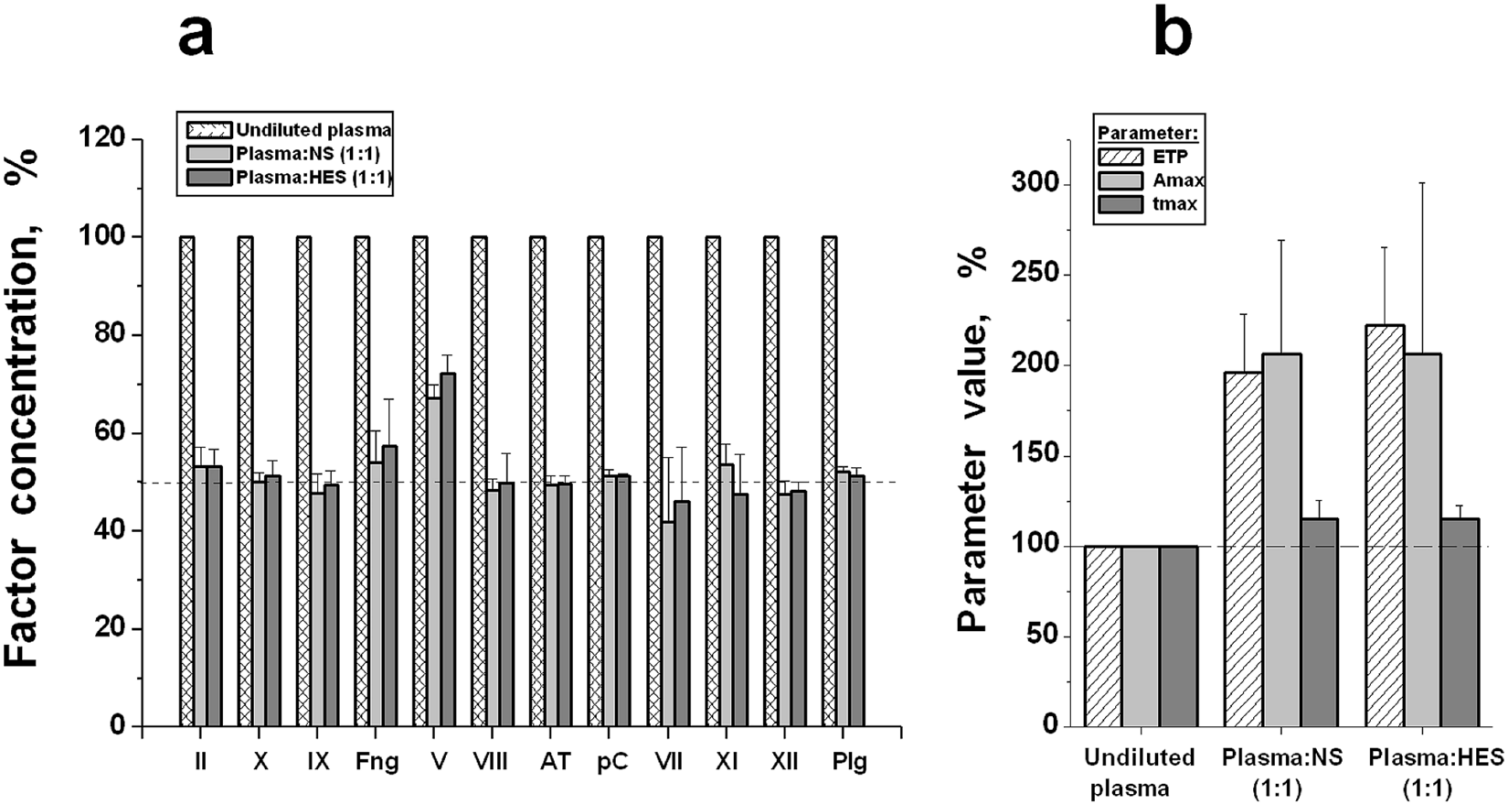
The decrease in concentration of coagulation factors and inhibitors during plasma dilution and its connection with coagulation efficiency. The results are presented in % relative to the corresponding value in undiluted plasma. (**a**) - Concentrations of the factors: II, X, IX, fibrinogen (Fng), V, VIII, VII, XI, XII; inhibitors: antithrombin III (AT) and protein C (pC); and plasminogen (Plg) in undiluted plasma, and plasma diluted two-fold with NS or HES. (**b**) - Parameters of TGT in these plasma samples: ETP (endogenous thrombin potential), A_max_ (maximal concentration of thrombin in the sample), and t_max_ (time to maximal thrombin concentration). The averaged values are presented as the mean values ± SD (n = 3).

Concentrations of coagulation factors and inhibitors were measured in undiluted plasma and after two-fold plasma dilution with NS and HES 130/0.4 for three different plasma samples. TGT was measured in parallel. The values of the initial factor concentrations were various in all the studied samples (two of fresh plasma and one of pooled frozen plasma (n = 4)); therefore, the concentrations of all the factors after dilution were calculated as the % relative to the initial concentration in each sample, which was taken as 100%. The obtained averaged results are presented in Fig. 4. As could be expected, all the concentrations decreased by approximately two times with good accuracy, excluding the factor V concentration, which decreased to a lower degree (Fig. 4a). The reason for this effect is now unclear. The parameters of TGT showed that thrombin generation was enhanced in spite of the identical decrease in the concentrations of factors and inhibitors of coagulation (Fig. 4b).

### Spatial dynamics of coagulation in plasma diluted with PEs *in vitro*

**Figure 5:**
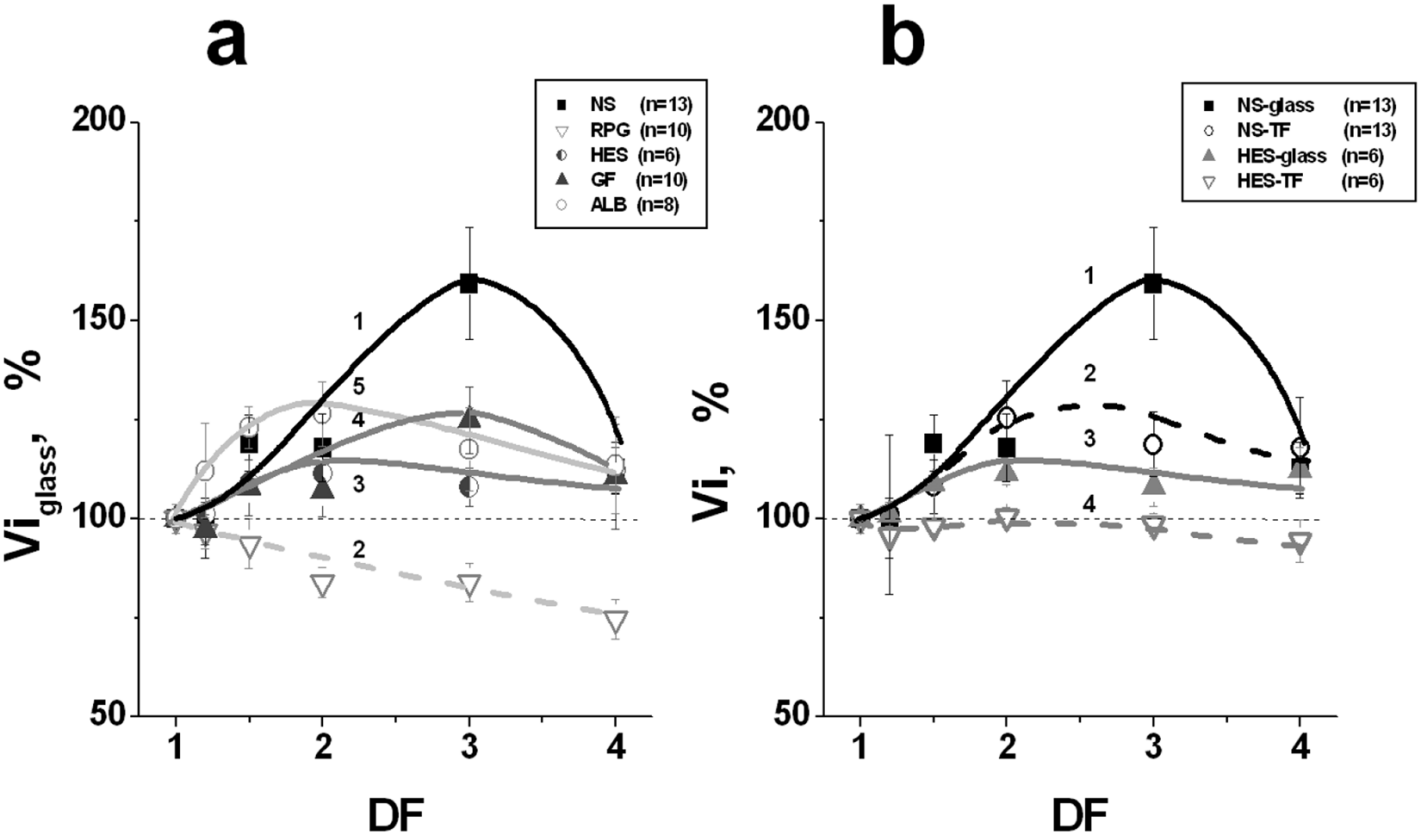
Initial clot growth rates at different levels of plasma dilution and under different types of coagulation activation. The results are shown in % relative to the corresponding rate in undiluted plasma. (**a**) – Clot growth rates in plasma diluted with: (1) – NS (n = 13); (2) – RPG (n = 10); (3) – HES (n = 6); (4) – GF (n = 10), and (5) – ALB (n = 8). Clotting was contact-activated. The difference between the starting undiluted plasma and the same diluted plasma was significant for all the PEs beginning from DF = 1.5 (ANOVA, *P* < *0.05*). (**b**) – Clot growth rates in plasma diluted with NS (curves of 1 and 2, n = 13), or HES (curves of 3 and 4, n = 6). Coagulation was activated by glass (curves of 1 and 3), or by TF (curves of 2 and 4). The mean values ± SD are shown. DF (the dilution factor) is equal to the ratio of the final volume of diluted plasma to its initial volume. The difference between the initial undiluted plasma and the same diluted plasma was significant beginning from DF = 1.5 for both types of activation in the case of NS, but only for contact activation in the case of HES (ANOVA, *P* < *0.05*). The difference observed between the two types of activation was significant at all DFs for HES, but only at DF = 3 for NS.

The spatial dynamics of coagulation in most cases also demonstrated acceleration with the plasma dilution. However, unlike ETP, the initial (Vi) and stationary (V) spatial rates of clot growth changed differently under plasma dilution with the various PEs. The corresponding averaged dependencies obtained for Vi under contact activation of coagulation are shown in Fig. 5a. The value of the maximal hypercoagulation effect in diluted plasma decreased gradually in this row of PEs: NS > ALB > GF > HES. For RPG, the value of Vi decreased with an increase of DF. The difference between the initial undiluted plasma and the same diluted plasma was significant for all the PEs beginning from DF = 1.5 (ANOVA, *P* < *0.05*).

**Figure 6:**
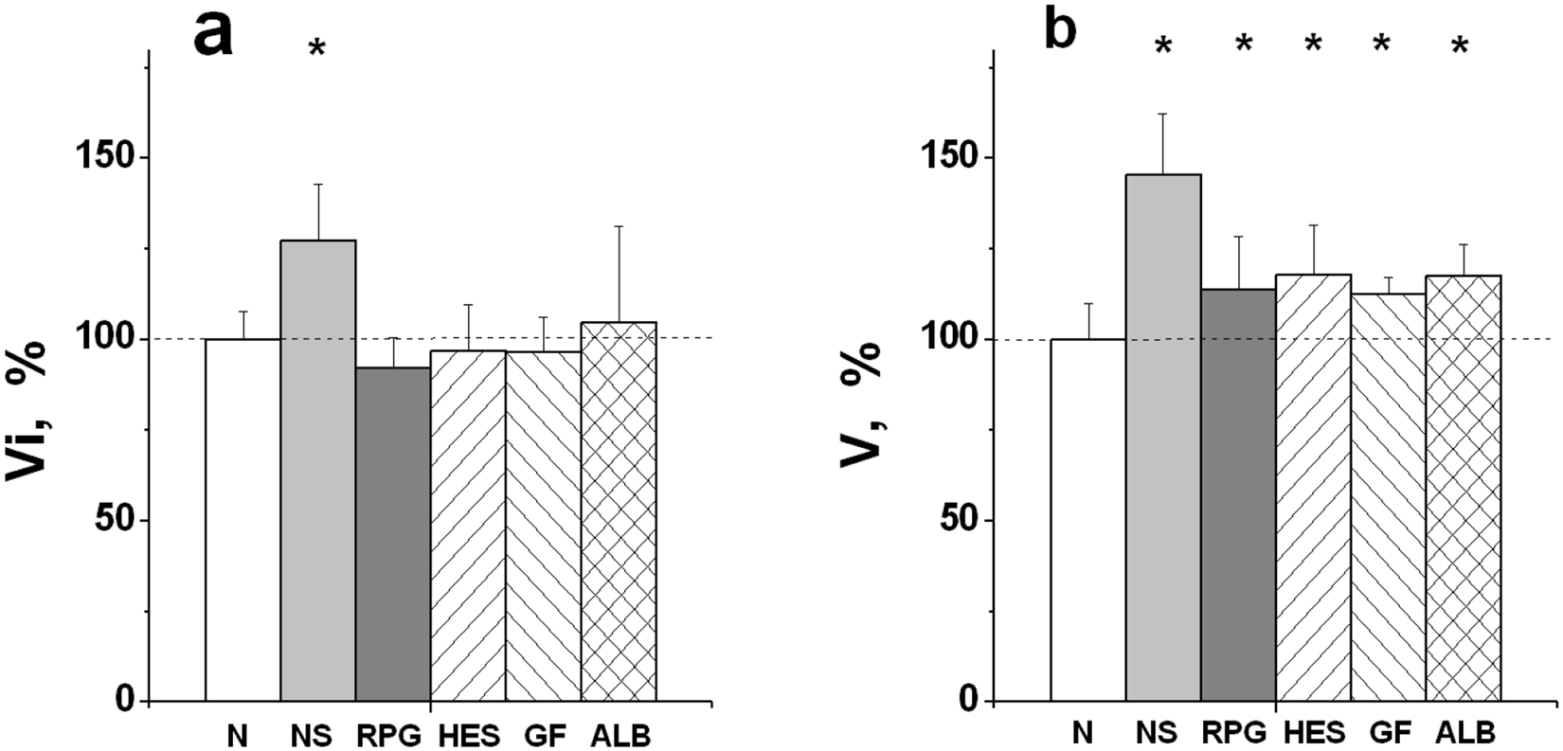
Clot growth rates in undiluted plasma (N) and in plasma diluted two-fold with various PEs. The initial (**a**) and stationary (**b**) spatial clot growth rates are presented. Coagulation was activated by TF (an extrinsic pathway). The values of Vi and V in undiluted plasma were accepted as 100%. The following PEs were used for dilution (see legend to Fig. 1): NS, RPG, HES, GF, and ALB. The mean values ± SD are presented. *Difference between undiluted and diluted plasma is significant (ANOVA and paired t-test of Student, *P* < *0.05*, n = 14 for ALB and n = 8 for all the other PEs).

A qualitatively similar picture was observed for two types of coagulation activation: using glass (an intrinsic pathway of activation), and using tissue factor (TF, an extrinsic pathway), but the values of the hypercoagulation shifts were always lower in the case of TF activation. The Vi values for dilution of plasma with NS and HES for the different types of coagulation activation are shown in Fig. 5b. The differences between initial undiluted plasma and the same diluted plasma were significant beginning from DF = 1.5 for both types of activation in the case of NS, but only for contact activation in the case of HES (ANOVA, *P* < *0.05*). The differences observed between the two types of activation were significant at all DFs for HES, but only at DF = 3 for NS. The averaged results characterizing the influence of various PEs on Vi and V in the case of extrinsic activation of clotting are presented in Fig. 6. In order not to overload this figure, only the averaged clot growth rates in undiluted plasma and plasma diluted with PEs twice are shown. The V values were significantly higher in diluted samples than in undiluted plasma for all investigated PEs (Fig. 6b). At the same time, Vi was increased significantly only in the presence of NS, but it remained within the limits of normal values for all the other solutions (in the case of RPG, there was a tendency towards a decrease of Vi, which, however, was not significant).

### Prevention of dilution-induced hypercoagulation with PEs containing inhibitors of thrombin

#### Impact of plasma dilution on coagulation in the presence of different concentrations of AT in the system

To test the hypothesis that a thrombin inhibitor can prevent hypercoagulation caused by dilution, experiments were carried out in which plasma was diluted with NS in the presence of a constant concentration of a natural thrombin inhibitor (AT). The different concentrations of AT were added to each sample of the series in the course of dilution, so that the final concentration of AT in each series of experiments was kept strictly constant under all degrees of plasma dilution.

**Figure 7:**
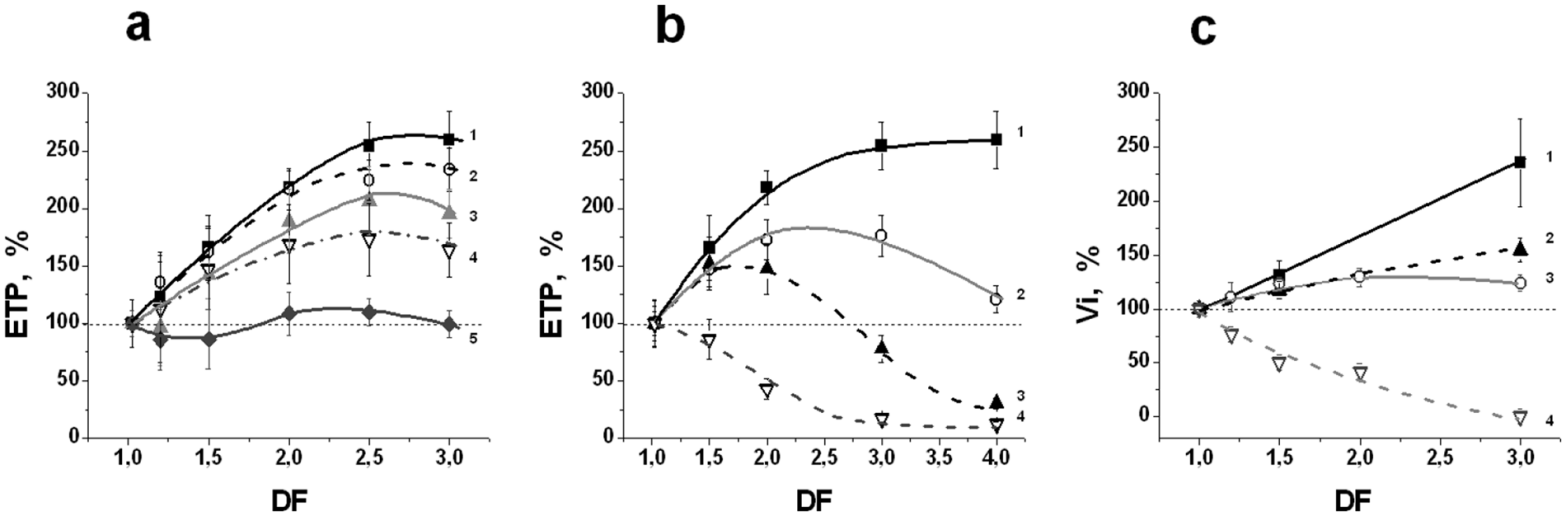
Thrombin inhibitors decrease dilution-induced hypercoagulation. The value of the corresponding parameter in undiluted plasma was taken as 100%. DF is a dilution factor. (**a**) - The increase of ETP during plasma dilution with NS decreases in the presence of AT. The final concentration of AT for each curve was constant and equal to (1) – 0; (2) – 0.145; (3) – 0.29; (4) – 0.58; and (5) – 0.87 IU/ml (n = 5–9). The ETP in diluted plasma differed from that in starting plasma in each series of experiments significantly beginning from DF = 1.5 for all the concentrations of AT, excepting 0.87 IU/ml (ANOVA, *P* < *0.05*). The difference between the curves with and without AT was significant for DF = 2.5, 3 (curve 2); DF = 2, 2.5, 3 (curves 3 and 4); and DF = 1.5, 2, 2.5, 3 (curve 5). (**b**) – Changes of ETP in plasma diluted with NS supplemented with different concentrations of the direct thrombin inhibitor HC-025s-IOC. The concentration of the inhibitor in PE was equal to: (1) – 0; (2) – 0.25; (3) – 0.5; and (4) – 1 µМ (n = 3). Coagulation was activated using TF (4 pM). The difference between the initial plasma and the same diluted plasma was significant for all DFs at any concentration of inhibitor (ANOVA, *P* < *0.05*). The difference between the curves in the presence and absence of inhibitor was significant for all the curves, excluding curves 2 and 3 at DF = 1.5. (**c**) - Initial clot growth rate (Vi) under plasma dilution with NS in the presence of different concentrations of thrombin inhibitors, and under the different methods of coagulation activation (n = 3 pools, each of 3 plasma samples). (1) - Contact activation (glass, n = 3); (2) - contact activation and AT in all the samples at the concentration of 0.87 IU/ml (n = 3); (3) – activation by TF (4 pM, n = 3); (4) – activation by TF and dilution with NS containing 8 µМ of the thrombin inhibitor HC-025s-IOC (n = 3). The mean values ± SD are shown. All the obtained differences were significant (α = 0.05 and statistical power of 80%) excluding the difference between curves 1 and 2 at DF = 1.5.

The values of ETP and Vi obtained in these experiments are presented in Fig. 7a and c, respectively. The value of hypercoagulation caused by dilution decreased in the presence of AT. The ETP in diluted plasma differed significantly from that in starting plasma in each series of experiments beginning from DF = 1.5 for all the concentrations of AT, excepting 0.87 IU/ml (ANOVA, *P* < *0.05*). Differences between the curves with and without AT were significant for DF = 2.5, 3 (curve 2); DF = 2, 2.5, 3 (curves 3 and 4); and DF = 1.5, 2, 2.5, 3 (curve 5). The difference in Vi between the initial plasma and the same diluted plasma were significant for all the curves and DF (Fig. 7c), but in a comparison of the paired curves with and without a thrombin inhibitor (1 and 2, or 3 and 4), the difference between corresponding curves was significant for all DFs in the case of curves 3 and 4, and only for DF = 3 in the case of curves 1 and 2.

#### The dilution of plasma with NS containing a low-molecular-weight direct thrombin inhibitor

The thrombin generation and spatial dynamics of clotting in plasma diluted with NS containing a new direct thrombin inhibitor HC-025s-IOC^35, 36^ are shown in Fig. 7b and c. This setting differs from the one presented in the previous section, since an inhibitor was put directly into the solution for plasma dilution. In this case, a higher degree of dilution led to a higher concentration of added inhibitor in the sample. This situation directly mimics the process that occurs *in vivo* during the introduction of various volumes of PE.

The results obtained with AT and the low-molecular-weight thrombin inhibitor were qualitatively similar. Hypercoagulation after dilution decreased with introduction of the thrombin inhibitor in NS. The value of this effect increased with the increasing concentration of thrombin inhibitor. All the differences between undiluted plasma and the same diluted plasma were significant for all the concentrations of inhibitor and DFs (ANOVA, *P* < *0.05*). The differences between all the curves with and without HC-025s-IOC (Fig. 7b,c) were significant.

## Discussion

A large number of works have studied the effects of haemodilution with PEs on haemostasis *in vitro* and *in vivo*. However, the results obtained are very contradictory.

The first studies led to the conclusion that PE should cause coagulopathy of dilution, since the classic tests of clotting (PT and APTT) in the course of haemodilution have always been prolonged^20, 25^. However, such prolongation was observed only when the concentration of procoagulant factors in the plasma decreased by 50–80%, suggesting that procoagulants are present in plasma in great excess^25, 26, 37^. The situation with anticoagulants (first of all AT) is different. Their reactions with the active factor-targets follow second-order kinetics, which means that the inhibition rate decreases in direct proportion to a decrease in the inhibitor’s concentration^28, 29^. We suppose that acceleration of clotting may be observed as the initial response of haemostasis upon the dilution of plasma up to ~2–2.5 times, since clotting should not be substantially suppressed due to a decrease in concentration of procoagulant factors in this DF range; however, at the same time, it must be accelerated because of a decrease in the concentration of anticoagulants. Combined with the possible development of hypercoagulation syndrome in trauma and subsequent DIC^38^; this shift in coagulation can lead to coagulopathy of consumption^39^ and, as a result, extensive bleeding.

Our assumptions described above are supported by works *in vitro* ^40–42^ and by a number of clinical studies on haemostasis after PE infusion^21–24^, which directly indicate an increase in the likelihood of thrombotic complications with increases in plasma dilution and the volume of transfused PEs. In this study, we addressed the question of how coagulation in platelet-poor plasma diluted with various PEs is affected by the fact of dilution. The pH and the concentration of Ca^+2^-ions in all the samples were kept constant.

It should be noticed primarily that the contemporary conception of haemostasis considers it to be a system in which blood cells take the most direct participation, especially the platelets^43^. In an activated state, they present their surface for the assembly of the tenase and prothrombinase complexes necessary for effective clotting^44^. The platelet concentration necessary for normal coagulation has been studied very extensively. In clinical guidelines, there are different thresholds for the minimal platelet concentration above which the transfusion of platelets is not necessary. The value of this threshold in different studies ranges from 10·10^3^ to 100·10^3^ cells/µl^6, 45, 46^. The last value is usually mentioned as a necessary threshold in special conditions, for example, during neurosurgery or when the activity of the patient’s platelets is decreased for some reason^46^. The most of the studies suppose that platelet concentrations should be 50·10^3^–100·10^3^ cells/µl^47^.

Not only does the concentration of all the coagulation factors and inhibitors decrease under haemodilution, the platelet concentration also decreases. That should make a contribution to a resulting effect of haemodilution in the body. However, it is important to note that at moderate degrees of blood dilution (by ~40–50%), a parallel decrease of the platelet concentration will virtually not affect coagulation (in the case when the initial platelet concentration is in normal range, i.e., above 175·10^3^–200·10^3^ cells/µl). In some studies, titration of the platelets’ effect on coagulation has been performed^48, 49^ (using measurements of clotting time or thrombin generation test, respectively). It was shown that for TGT, the dependency of clotting efficiency on the platelet concentration constitutes a steep curve, which gradually reaches up to the maximum level (a view of the Michaelis-Menten curve). Moreover, a decrease of coagulation rate, directly proportional to the decrease of the platelet concentration, is observed only at the initial part of the curve (from 0 to 10·10^3^ cells/µl). At concentrations above ~50·10^3^ cells/µl, the efficiency of coagulation ceased to depend on the platelet concentration^49^. This suggests that with moderate dilution of blood when the cell concentration does not usually drop under the plateau (providing maximum efficiency of coagulation), the observed effects of haemodilution are related to the dilution of plasma coagulation factors and inhibitors but not to the reduction in the platelet concentration. At higher dilutions of blood, platelets will make a contribution to the resulting effect of haemodilution (slowing down the rate of coagulation). At high degrees of decrease in the platelet concentration, the MA parameter in TEG should also decrease since it is determined primarily by the concentrations of platelets and fibrinogen^50^.

Thus, our investigation of coagulation using dilution of platelet-poor plasma reflects the main impact of the dilution process on coagulation at moderate degrees of dilution (if other factors such as hypothermia, the presence of inflammation, the wound surface, decreased function of platelets or coagulation diseases, etc. are absent).

In accord with the literature data, APTT and PT were prolonged in the diluted plasma (Fig. 1a,b), supporting the widely accepted hypothesis that dilution impairs clotting by reducing the concentration of procoagulant factors. However, this fact cannot serve as a direct proof of this hypothesis because the PT and APTT tests are carried out in conditions of high baseline dilution (3-fold) using the highest activation of coagulation and under complete saturation of the system with the phospholipid surface. By their nature, both tests have low sensitivity towards the hypercoagulation state^51^. They measure the maximum clotting activity (at dilution degrees >3), which is limited only by the concentrations of the procoagulant factors in the sample. These concentrations decrease under further dilution of plasma, resulting in APTT and PT prolongation. However, during real clotting (without superfluous high dilution, activation or a phospholipid surface), a shift to the hypercoagulation state may occur in the system upon dilution, which may be related primarily to the slowing down of the reactions of haemostasis inhibition due to the dilution of coagulation inhibitors. These kinetic shifts to hypercoagulation cannot be registered by measuring only the highest possible level of coagulation (PT and APTT).

As expected, the other clotting tests, such as TT and RT, which in contrast to the PT and APTT are performed at lower dilution degree (2-fold), showed decreased values for the dilution of plasma up to 3-fold with any PE. Thus, in general, the results obtained suggest the possibility of enhanced clotting at moderate degrees of plasma dilution.

To reliably detect both hypo- and hypercoagulation states, integral coagulation tests should be used. The vast majority of studies of coagulation in the presence of haemodilution have used thromboelastography (TEG) in whole blood^23, 41, 52–56^. However, the dilution of blood often changes the different parameters of this test in opposite directions, shortening the reaction time (r) and coagulation time (k), which indicates the acceleration of clotting but decreasing simultaneously the maximal clot firmness (MA or G), which is considered a weakening of coagulation. The decreasing in MA is connected first of all with the decrease in concentrations of fibrinogen and platelets^50^. Thus, the data obtained by TEG does not look quite apparent. In this work, we used cell-depleted plasma and two other global coagulation tests: the TGT^57, 58^ and the test of TD (measurement of the clot growth rate in space)^59^. Both methods use lower activation of coagulation than the standard clotting tests, with an activation level close to the activation that occurs in the body. Each of them is highly sensitive to both hypo- and hypercoagulation disorders of clotting^51, 60, 61^.

Plasma dilution by PE of any type always increased thrombin generation (Figs 2 and 3). The effect varied widely from plasma to plasma, but it was qualitatively the same for all the PEs tested. This may indicate that the increase of thrombin generation under dilution is associated not only with the properties of the specific PE but also with the process of plasma dilution itself.

In our experiments, the equal decrease (in %) in concentration of procoagulant factors and inhibitors at 2-fold dilution of plasma using any PE (NS or HES 130/0.4) led to increased coagulation (Fig. 4). All the above-mentioned results support our hypothesis. At moderate degrees of dilution, coagulation is more sensitive to the decrease in plasma concentration of inhibitors than procoagulant factors. The obtained results are in good agreement with the results of a number of published studies^23, 41, 52–56^.

The existence of dilution-induced hypercoagulation was also confirmed using the test of TD. The Vi rates for all the PEs (excluding RPG) increased at moderate degrees of plasma dilution (Fig. 5a). The magnitude of the hypercoagulation effect depended on the PE type and the pathway of coagulation activation. The effects were more pronounced with the contact pathway of activation (Fig. 5b). Similar effects were observed in earlier work^48^ where the influence of plasma dilution on coagulation was investigated (based on measurements of coagulation time in plasma) after activation using different methods: by thromboplastin, glass particles, or asbestos fibres. The underlying cause is not evident at the moment. However, the decrease in the concentration of not only АТ but also other coagulation inhibitors, for example TFPI^42^, can be important for a hypercoagulation shift upon dilution. Hence, it is possible to assume that Vi (and V) are dependent on the concentration of inhibitors of contact activation, which are also decreased upon dilution.

In the case of extrinsic clotting activation, the dilution weakly affected Vi, while V was significantly increased for any of the studied PEs (Fig. 6b).

The results obtained by TD suggest that unlike NS, colloids may have a dual effect on clotting. On the one hand, the dilution of plasma with any colloidal solution, like its dilution with NS, causes acceleration of thrombin generation due to a change in the balance of the pro- and anti-coagulant properties of the system. On the other hand, the colloids may obviously have their own effects on the formation of a fibrin clot and thereby may inhibit the rate of spatial clot growth. In addition, the inhibitory effect of the various colloidal solutions may vary greatly. For example, it was shown using TEG that the effect of albumin during haemodilution is obviously associated only with dilution of the plasma, whereas the colloids, such as GF and HES, worsened the quality of the clot and slowed down the process of its formation. However, the data from these studies cannot explain an increase in V in the course of plasma dilution *in vitro* with various PEs.

To prove that the dilution-induced hypercoagulation was due to the reduced concentrations of coagulation inhibitors (primarily AT), we carried out two sets of experiments. In the first series, plasma was diluted with NS, but the concentration of AT in all the samples remained constant. In the second series, plasma was diluted with NS that additionally contained one of our new direct low-molecular-weight synthetic thrombin inhibitors. It was shown earlier that this new inhibitor effectively reduced the ETP and Vi (as well as V) in plasma in a concentration-dependent manner^36^.

In both lines of experiments, we observed a partial correction of hypercoagulation caused by dilution. The value of the effect depended on the concentration of the inhibitor in the system. The concentration of AT of 1 IU/ml may be considered optimal, since at this concentration, the values of ETP and Vi are the closest to their values in normal undiluted plasma (Fig. 7). The concentrations of the synthetic inhibitors that can be used in PE depend on the equilibrium constants of binding of these compounds with thrombin and the other proteins in plasma. They should be selected separately for every inhibitor.

Thus, haemodilution affects haemostasis in many ways. The total effect depends on many parameters such as the degree of dilution; the type of PE; the method used to estimate the status of coagulation; the time from haemodilution to when the measurement of haemostasis status was carried out, etc. Sometimes the constant levels of рН and/or Ca^+2^-ions are not supported in *in vitro* experiments. This leads to diverse conditions of coagulation after plasma recalcification at various degrees of plasma dilution. This is why contradictions exist in the modern scientific literature on the question of how haemodilution affects coagulation.

The results obtained by us *in vitro* confirmed the existence of hypercoagulation conditions upon plasma dilution with various PEs up to ~2–2.5 times. This hypercoagulation is initially caused by a decrease in the concentrations of coagulation inhibitors in diluted plasma, since the addition of thrombin inhibitors to plasma or PEs partially prevents the hypercoagulation shifts caused by dilution. A further increase in the dilution (more than 3–4 times) causes hypocoagulation because of the pronounced decrease in the concentrations of procoagulant factors.

The limitation of our work is that we investigated *in vitro* only one mechanism included in the development of clotting disorders upon haemodilution: the impact of the very process of plasma dilution on coagulation status. The clinical relevancy of this process should be clarified in *in vivo* studies. It is necessary to understand what factors are basic in the formation of clots after haemodilution *in vivo* and how various colloids may change the quality of clots. This may be, in the end, the most important factor for the clinical application of each PE. All these questions undoubtedly require further study.

## Methods

Detailed description of the reagents used may be found in Supplementary Information.

APTT, PT, TT, RT and TGT were performed in platelet-poor plasma (РРР). The clot growth rates were measured in platelet-free plasma (PFP). All methods were carried out in accordance with the approved guidelines.

### Ethics Statement

This study was approved by the Ethical Committee of the Center for Theoretical Problems of Physicochemical Pharmacology (as part of the program «Investigation of the Pathologic and Physiologic Mechanisms of Haemostasis» (Permit Number: 21-04-2009)). All participants provided written informed consent before blood collection. Blood was received at the station of blood transfusion (National Research Center for Hematology, Moscow, Russia) and was used without authentication.

### Plasma preparation

Plasma for analysis was prepared from the blood samples collected in standard Vacuette tubes with 3.8% (0.129 М) sodium citrate in a volume blood : citrate ratio = 9 : 1. РРР was prepared via centrifugation of blood for 15 min at 1600 g. PFP was obtained using an additional centrifugation of РРР for 5 min at 10,000 g (at ambient temperature).

### Additional processing of plasma expanders

To preserve the standard conditions of recalcification in citrate plasma diluted with PEs to varying degrees, the concentration of citrate in all the samples was kept constant, and recalcification was performed always with the same amount of added CaCl_2_. Furthermore, in all the samples, the same pH value (7.4) was maintained. To achieve the above-described conditions, CaCl_2_ was added to PE up to the concentration of 1.5 mM. This corresponded to the average concentration of free calcium ions in the blood plasma. Then, a solution of 3.8% sodium citrate (pH 7.5) was added. The PE : citrate ratio was equal to 9 : 1, which corresponds to the standard conditions of blood collection. Thus, the concentration of free calcium ions, and of citrate in PE and undiluted citrated plasma, were approximately similar and did not change significantly under different degrees of dilution of this plasma with PEs. To maintain a constant pH in PE, a HEPES solution (10 μl of a 2 M solution per 1 ml of PE, pH 7.4) was added. Before the experiments, pH was measured in all the PEs, and if necessary, its value was adjusted to 7.4.

In some of the experiments, thrombin inhibitors (AT or HC-025s-IOC) were added to NS at various concentrations (to 5% of the total volume of PE).

The degree of dilution of plasma (a dilution factor, DF) was characterized by the ratio of the plasma volume after dilution to its initial volume (DF = V_diluted plasma_/V_initial plasma_). Undiluted plasma (DF = 1) and plasma diluted with PEs 1.2, 1.5, 2, 2.5, 3 and 4 times (in separate experiments up to 4.5 times) were used in the experiments. For preparation of such samples 0, 0.2, 0.5, 1, 2, and 3 (or 3.5) volumes of PE were added to 1 volume of undiluted plasma, respectively. The degrees of plasma dilution (in %) in these samples were equal to 0, 16.7, 33.3, 50.0, 66.7, and 75%.

### Standard clotting times

APTT, PT, RT, and TT were measured by standard methods according to manufacturers’ instructions using aggregometer Biola (Moscow, Russia). The final volumes of the samples for each test were as follows:

APTT: 100 μl of the plasma sample + 100 μl of the activator (erylid + 0.5% kaolin) + 100 μl of CaCl_2_ (25 mM);

PT: 100 μl of the plasma sample + 200 μl of the mixture (100 μl thromboplastin (ISI 1.2–1.4) + 100 μl of CaCl_2_ (25 mM));

TT: 100 μl of the plasma sample + 100 μl of the thrombin solution (an activity of thrombin was selected to give TT in undiluted plasma of 15 sec);

RT: 100 μl of the plasma sample + 100 μl of CaCl_2_ (25 mM).

All the samples were measured in triplicates.

### Thrombin generation assay

Kinetics of thrombin generation in plasma was measured using a modification of the standard method^57, 58^. The original method supposes a dilution of initial plasma sample 1.5 times in the course of measurement. This additional dilution of the plasma at measurement may distort accuracy of the results obtained in conditions of dilution. Therefore we modified a procedure as described in^36^ to avoid additional dilution of the sample during measurement. Thrombin-specific fluorogenic substrate BOC-Ile-Gly-Arg-AMC^59^, where ВОС is the tret-butoxicarbonyl residue and АМС is 7-amino-4-methylcoumarine residue, was synthesized in the Institute for Medical and Biological Chemistry (Moscow, Russia). Measurements were executed as follows. PPP was placed in the wells of a 96-well flat-bottom microtiter plate (200 μl/well). Thereafter, 2 μl of the fluorogenic substrate solution in DMSO (41 mM) was added, and the samples were incubated at 37 °C for 3–5 min before being activated with 3 μl of an activator. Twenty-fold diluted rabbit thromboplastin solution (PT reagent, Renam, Moscow, Russia) in buffer containing 20 mM of HEPES (pH 7.5), 140 mM of NaCl, and 1.4 M of СаСl_2_, was used as activator.

Final concentrations of reagents in each sample were as follows: fluorogenic substrate, 400 µM; tissue factor, 4 pM; added CaCl_2_, 20.5 mM. The accumulation of the fluorescent reaction product (7-amino-4-methylcoumarin, AMC) continuously registered at 37 °С using an fluorometric Fluoroscan II reader (LabSystem, Helsinki, Finland) (λ_excitation_ = 380 nm; λ_emission._ = 440 nm). Each result is the averaged value of two parallel measurements.

Data analysis was carried out using Origin 6.0 software (Microcal Software, Northampton, MA, USA). Fluorescence signal was calibrated by the fluorescence value of 1 µM of AMC in the given plasma (in the presence of the fluorogenic substrate). Thrombin concentrations at each time point were calculated using previously determined kinetic constants of thrombin with respect to used substrate (К_М_ = 62 μM and k_cat_ = 57 min^−1^)^59^. Corrections were made for the nonlinear fluorescence dependence on AMC concentration («inner filter» effect), substrate consumption during reaction, and the contribution of the thrombin-α_2_-macroglobulin complex’s work into the total signal of measured fluorescence^60^.

### Thrombodynamics assay (measurement of the clot growth rates)

TD is the new integral test for the assessment of haemostasis efficiency. It measures a spatial clot growth rate in a thin layer of plasma without stirring where coagulation is launched from the surface of an activator that is strictly localized in space. A clot size versus time was determined by measuring plasma light scattering in the area of clot formation. The TD test was performed in PFP according to previously described method^61–63^ using reagents and a device that were developed by HemaCore LLC (Moscow, Russia).

Spatial dynamics of coagulation was characterized by two parameters of the TD: initial (Vi) and stationary (V) spatial rates of clot growth. Both, an intrinsic or extrinsic pathway for activation of clotting were used (by glass, or using the surface covered by an immobilized tissue factor (TF), respectively). Only the Vi value could be measured in the contact activated plasma, because of the strong spontaneous coagulation (i.e., coagulation, not directly concerned with the activator’s surface, was sharply accelerated in this diluted plasma samples, and the spontaneous clots filled up the entire area of the sample faster than a stationary level for the rate was achieved). At the activation using an extrinsic pathway all the measurements were carried out in the presence of an inhibitor of contact activation (corn trypsin inhibitor, CTI). This reduced a level of spontaneous coagulation and allowed us to determine the both Vi, and V.

### A protocol for the TD measurement

The PFP sample (120 µl) was mixed (only in the case of activation by TF) with the dry reagent of the contact-activation inhibitor (corn tripsin inhibitor), and incubated at 37 °С for 15 min. The sample was then recalcified by mixing with the dry calcium acetate reagent, and placed into a plastic chamber. The experiment was launched by the introduction of an activator-plate into this chamber. A glass plate or plate, the end of which was covered with immobilized tissue factor^64^ was used for clotting activation. The sample was constantly photographed for 30 min (6 sec/frame).

The results were processed using a specially developed by manufacturers software using an automated calculation algorithm. The curve clot size/time was used to measure the Vi and V (as slopes on the segments of 2–6 min and 15–25 min from clot growth start for the Vi and V, respectively). Each result is the averaged value of two parallel measurements.

### Measurement of the concentrations of coagulation factors and inhibitors

These measurements were carried out for undiluted plasma and plasma after two-fold dilution with NS or HES 130/0.4 by using an optical coagulometer (Instrumentation Laboratory ACL TOP 700, Bedford, MA, USA) and corresponding methods and reagents from manufacturers.

### Statistical analysis

The normality of distribution for all the measured coagulation parameters was examined beforehand using the D’Agostino-Pearson test in the program MedCalc Statistical Software bvba (version 14.12) (Belgium). It was shown that APTT, PT, RT, TT, as well as ETP, A_max_, t_max_, t_lag_ (in TGT), and Vi and V (in the TD) were normally distributed. The results were presented as the mean values ± standard deviation (SD). In the presence of normal distribution, the significance of difference between the groups of diluted, and undiluted plasma was assessed using one-way ANOVA and Student’s paired t-test. The difference was considered significant at *P* < *0.05*. Both tests showed similar results. The statistical power for comparison of the parameters of undiluted and diluted plasma was ≥80% for all the DFs beginning from DF = 1.5.

## Electronic supplementary material

Reagents
