# Supplementary material for: Moderate plasma dilution using artificial plasma expanders shifts the haemostatic balance to hypercoagulation
Source: Sci Rep. 2017 Apr 12;7:843. doi: 10.1038/s41598-017-00927-w (PMC5429808; doi:10.1038/s41598-017-00927-w)
Supplement: Supplementary file 1 — Reagents [file 41598_2017_927_MOESM1_ESM.pdf]

SUPPLEMENTARY INFORMATION

Moderate plasma dilution using artificial plasma expanders shifts the haemostatic balance to hypercoagulation

Elena I. Sinauridze, Alexander S. Gorbatenko, Elena A. Seregina, Elena N. Lipets, Fazoil I. Ataullakhanov

Reagents

The following reagents were used: the reagent kits «Coagulo-test», «Diagem P», «Thrombin-test», and thromboplastin (ISI 1.2-1.4) manufactured by Renam (Moscow, Russia); the slow thrombin-specific fluorogenic substrate BOC-Ile-Gly-Arg-AMC [56] synthesized in the Scientific Research Institute of Biomedical Chemistry (Moscow, Russia); 7-amino-4-methylcoumarin (AMC), $CaCl_2$, and NaCl (Sigma-Aldrich, St. Louis, MO, USA); dimethyl sulfoxide (DMSO) (Merck Chemicals, GmbH, Darmstadt, Germany), and 4-(2-hydroxyethyl)-1-piperazine-2-ethanosulphonic acid (HEPES, Fisher Biotech, Fair Lawn, NJ, USA). Corn tripsin inhibitor (CTI) and antithrombin III (AT) were isolated from corn and human plasma in the Institute of Protein (Pushchino, Russia) and National Research Center for Hematology (Moscow, Russia), respectively. Low-molecular-weight direct thrombin inhibitor HC-025s-IOC[32] was synthesized in the Institute of Organic Chemistry RAS (Moscow, Russia). For the measurements of thrombodynamics the corresponding kits and consumables were used manufactured by HemaCore LLC (Moscow, Russia). Clotting in the thrombin generation test (TGT) was activated with solution of thromboplastim. Glass or thromboplastin immobilized on the surface of a plastic plate was used as activators of coagulation in the test of thrombodynamics. The concentration of the tissue factor (TF) in the solution and on the surface of the activator was measured using Actichrome® TF test (American Diagnostica, Stamford, CT, USA).

The following standard plasma expanders (PEs) were used: NS - physiological solution (normal saline, Medical holding «Juno» LLC Medsintez, Russia); RS - Ringer's solution (saline solution containing: 147 mM Na^+; 4 mM K^+; 2.25 mM Ca^{+2} and 155.6 mM Cl^-, Hemofarm A.D., Vrshach, Serbia); HES 130/0.4 - 6% solution of hydroxyethyl starch with molecular mass of 130000 D and a degree of substitution by the hydroxyethyl groups of 0.4 (Voluven, Fresenius Kabi, Bad Homburg, Germany); RPG - 6% solution of dextran with molecular mass of 30000-40000 D (Reopolyglukin, Biochemist LLC, Saransk, Russia); ALB - 10 % solution of human albumin (Nizhny Novgorod Regional Blood Transfusion station named after N.Y. Klimova,

Nizhny Novgorod, Russia); GF – 4% solution of cuccinilated gelatin with molecular mass 30000 D (Gelofusine, B. Braun Melsungen A.G., Germany).
